# Risk factors for community-acquired pneumonia in children under five years of age in the post-pneumococcal conjugate vaccine era in Brazil: a case control study

**DOI:** 10.1186/s12887-016-0695-6

**Published:** 2016-09-22

**Authors:** Eduardo Jorge da Fonseca Lima, Maria Júlia Gonçalves Mello, Maria de Fátima Pessoa Militão de Albuquerque, Maria Isabella Londres Lopes, George Henrique Cordeiro Serra, Debora Ellen Pessoa Lima, Jailson Barros Correia

**Affiliations:** 1 Instituto de Medicina Integral Prof. Fernando Figueira(IMIP), Recife, PE Brazil; 2 Faculdade Pernambucana de Saúde, Recife, PE Brazil; 3 Centro de Pesquisas Aggeu Magalhães – Fiocruz Pernambuco, Recife, PE Brazil; 4 Universidade Federal de Pernambuco, Recife, PE Brazil; 5 Universidade de Pernambuco(UPE), Recife, PE Brazil

**Keywords:** Risk factors, Pneumonia, Children, Pneumococcal vaccines, Influenza vaccines

## Abstract

**Background:**

Pneumonia plays an important role in children’s morbidity and mortality. In Brazil, epidemiological and social changes occurred concomitantly with the universal introduction of the 10-valent pneumococcal conjugate vaccine. This study identified risk factors for pneumonia following the implementation of a pneumococcal vaccination program.

**Methods:**

A hospital-based, case-control study involving incident cases of pneumonia in children aged 1–59 months was conducted between October 2010 and September 2013 at a tertiary hospital in northeastern Brazil. The diagnosis of pneumonia was based on the World Health Organization (WHO) criteria. The control group consisted of children admitted to the day-hospital ward for elective surgery. Children with comorbidities were excluded. The risk factors for pneumonia that were investigated were among those classified by the WHO as definite, likely and possible. A multivariate analysis was performed including variables that were significant at *p* ≤ 0.25 in the bivariate analysis.

**Results:**

The study evaluated 407 children in the case group and 407 children in the control group. Household crowding (OR = 2.15; 95 % CI, 1,46–3,18) and not having been vaccinated against the influenza virus (OR = 3.59; 95 % CI, 2,62–4.91) were the only factors found to increase the likelihood of pneumonia. Male gender constituted a protective factor (OR = 0.53; 95 % CI, 0,39–0,72).

**Conclusion:**

Changes on risk factors for pneumonia were most likely associated with the expansion of the vaccination program and social improvements; however, these improvements were insufficient to overcome inequalities, given that household crowding remained a significant risk factor. The protection provided by the influenza vaccine must be evaluated new etiological studies. Furthermore, additional risk factors should be investigated.

## Background

Community-acquired pneumonia (CAP) is a major public health issue and a principal cause of morbidity and mortality in children under 5 years of age [1]. According to the World Health Organization (WHO) estimates, 156 million new cases of pneumonia occur annually worldwide in children under five, with 95 % of these cases occurring in developing countries [1, 2]. CAP has been associated with various child-related and environmental risk factors that contribute to increasing the incidence and/or severity of the disease [3, 4].

Children living in remote regions where poverty and hunger are severe are most susceptible to pneumonia [1, 5]. In general, the poorer access of less privileged families to healthcare services means that they delay seeking adequate care, resulting in the deterioration of their condition and an increased risk of hospitalization [1, 6, 7].

Although a reduction in all-cause of child mortality occurred worldwide between 2000 and 2011, including a reduction in pneumonia-related deaths, in 2011, CAP was estimated to account for more than one million child deaths, 80 % of which occurred in children under 2 years of age [1, 8].

Approximately 50 conditions have been described in the literature that, if present, may increase the risk of developing pneumonia [3, 4, 9]. The WHO classifies the risk factors for CAP in children living in developing countries as definite, likely or possible [2]. A recent systematic review with meta-analysis evaluated the quality of evidence and the strength of the association between 19 risk factors and severe acute lower respiratory tract infection in children under five [10]. In the studies evaluated, seven risk factors were shown to be significantly associated: low birth weight, undernutrition, household air pollution, human immunodeficiency virus (HIV) infection, non-exclusive breastfeeding, household crowding and incomplete immunization.

In Brazil, the measles vaccine has been included in the immunization program for over 40 years. In 1999, universal vaccination against *Haemophilus influenzae* type b (Hib) was introduced, contributing to a reduction in the number of cases of pneumonia associated with Hib [11, 12]. Introduction of the 10-valent pneumococcal conjugate vaccine (PCV10) in 2010 has likely reduced the incidence of CAP even further [13, 14]. For several years, the anti-influenza vaccine, which is also associated with pneumonia prevention [15], was offered to 6-to 24-month-old children during periods when the incidence of the disease is higher; however, in 2014, its use was extended to children up to 5 years of age [16].

Studies on risk factors for CAP have been performed in Brazil for several years [3, 17–19]. Nevertheless, the socioeconomic changes occurring in the country in recent years [20], together with the expansion of the national childhood immunization program, may have caused changes in these factors; however, this issue remains to be clarified.

The presence of comorbidities as risk factors for CAP in childhood has been well defined in previous studies [21], and these comorbidities are accepted as the main risk factors. In our study, we intended to verify risk factors in previously healthy children.

Therefore, the objective of the present study was to analyze the risk factors for community-acquired pneumonia in children under 5 years of age, excluding neonates.

## Methods

A hospital-based, case-control study involving incident cases of pneumonia was conducted between October 2010 and September 2013 at the *Instituto de Medicina Integral Prof. Fernando Figueira* (IMIP) in the city of Recife, Pernambuco, northeastern Brazil. One control was selected for each case. This teaching hospital provides healthcare to users of the Brazilian National Health Service (SUS) only. According to data from the National Health System (DATASUS) [22], IMIP admitted approximately 30 % of all children under five with pneumonia in the state of Pernambuco during 2012.

Overall, the study included children from 1 to 59 months of age for whom a vaccination card was available. Children were excluded if they had any concomitant primary disease, such as heart, liver, or kidney disease;chronic lung disease;neuropathy; hemoglobinopathies;congenital lung malformation; or a known diagnosis of immunodeficiency.

The case group consisted of children admitted to the hospital wards or to the intensive care unit with a clinical and radiological diagnosis of CAP made in accordance with the following WHO diagnosis criteria: 1) increased respiratory rate (rate >60 breaths/minute if aged <2 months, >50 breaths/minute if aged 2–11 months, and >40 breaths/minute if aged 12–59 months); 2) lower chest wall in drawing (severe pneumonia); or 3) cyanosis and/or inability to feed or drink (very sever pneumonia) [23]. Chest X-rays were analyzed independently by two radiologists according to the WHO criteria for epidemiological studies on vaccine effectiveness [24].

To render the study feasible, a control group was selected from among members of the community served by the hospital. This group consisted of healthy children who had been admitted to the hospital for elective surgery and didn’t have previous history or diagnosis of pneumonia by WHO criteria at the time of recruitment. The controls were recruited in accordance with the eligibility criteria on the same day or within 3 days of case allocation.

The main variables evaluated in the study are summarized in Table 1, including the definition and categorization of each variable. We included child-related factors (birthweight, breastfeeding, nutritional status, previous respiratory disease and/or allergy, previous hospitalization, 10-valent pneumococcal conjugate vaccine, influenza vaccine) and sociodemographic factors (maternal education, household crowding, basic sanitation, maternal age, smoking in the home and maternal smoking, monthly family income) [10, 25, 26]. Parents or guardians answered a specific questionnaire. To minimize issues such as missing data and biases, the team of investigators collected data as soon as possible after the admission of each case. The study team followed up all patients throughout their hospitalization period.

**Table 1:** Risk factors for pneumonia as defined in the study methodology

Sociodemographic factors	Maternal education	1) Did not finish high school (<11 years of schooling) 2) Finished high school (≥11 years of schooling)
	Household crowding	Defined as ≥2 individuals sleeping in the same room as the child.
	Basic sanitation	The disposal of bathroom/toilet waste in the household was classified according to whether a public sewage system/septic tank was present.
	Maternal age	Based on the WHO concept of adolescence, maternal age was categorized into <19 years and ≥19 years.
	Smoking in the home and maternal smoking	Whether any members of the household smoked and whether the child’s mother smoked.
	Monthly family income	Total household income, i.e., considering the earnings of all individuals living in the household, categorized as ≤1 or >1 minimum wage. (The minimum wage is the lowest remuneration that employers may legally pay to workers per month. During the period corresponding to the data collection the range was U$ 210.00 to 230.00)
Child-related factors	Birthweight	A continuous numeric variable, recorded in grams, either obtained from the vaccination card, certificate of live birth, medical records or as reported by the child’s mother. Categorized as low (<2500 g) or normal birth weight.
	Prematurity	Preterm is defined as babies born alive before 37 weeks of pregnancy are completed. This information was expressed as a dichotomous ordinal variable.
	Exclusive Breastfeeding	The child’s mother or guardian was asked if she had exclusively breastfed the child during 4 months. This information was expressed as a dichotomous variable.
	Nutritional status	Assessed according to the WHO child growth standards [26] using z-score calculations. According to a-2 z-score, the weight-for-age ratio was classified into two categories: very low or low weight for age and normal weight for age.
	Previous respiratory disease and/or allergy	This was a nominal categorical variable obtained by the investigator, defined by the presence of asthma/wheezing, coughing for more than 1 month, tuberculosis, eczema, rhinitis or other conditions, as reported by the child’s parent/guardian.
	Previous hospitalization	The child’s mother or guardian was questioned regarding whether the patient had been previously hospitalized for any reason.
	10-valent pneumococcal conjugate vaccine	With respect to the pneumococcal conjugate vaccine, children were considered to have been vaccinated if they had received at least two doses prior to their first birthday or one dose after their first birthday [25].
	Influenza vaccine	We considered that children who were immunized according to the immunization card received: a) at least one dose if they received the prime vaccination previously and had been immunized with two doses Or b) two doses of the prime vaccination Children under 6 months of age and those over 6 months of age who had not been immunized were classified as unvaccinated.

For sample size calculation, we used STATCALC (EpiInfo 6.04d), with the following parameters: estimated rate of 10 % of children unvaccinated with pneumococcal vaccine in the control group, 90 % statistical power to detect an odds ratio (OR) equal to 2.0, with an alpha error of 5 % and a proportion of 1 case for 1 control. The sample size obtained was 804 patients (402 cases and 402 controls). Due to possible losses we collected data from 452 cases and 407 controls. At the analysis we generated a list of random numbers in order to have one case to one control (407 at each group).

Data regarding cases and controls were organized in an Excel® spreadsheet (Microsoft® Windows) and submitted to a consistency check. In the exploratory analysis, we evaluated several cut-off points, and the variables were then dichotomized to enable a comparison with similar studies. In the bivariate analysis, odds ratios were calculated using the chi-square test or Fisher’s exact test as appropriate. The Statistical Package for Social Sciences software program, version 20.0, was used throughout the statistical analysis. Risk factors reaching a significance level of *p* ≤ 0.25 in the bivariate analysis were then included in a multivariate analysis. In the multivariate analysis, which was conducted in accordance with the backward selection method, the significance level was defined as *p* ≤ 0.05 [27].

This study protocol was submitted to the internal review board of the *Instituto de Medicina Integral Prof. Fernando Figueira,* approved under protocol #1860 and registered under approval number 0128.0.099.000-10. All parents or guardians signed an informed consent form.

## Results

The study included 814 children: 407 cases and 407 controls. Frequency distribution of the variables related to the child’s past and current health, comparing cases and controls, and the results of the bivariate analysis are described in Table 2. There were proportionally more male children and more children over 1 year of age in the control group. In the bivariate analysis, the variables that were significantly different between groups were age < 1 year, low/very low weight for age, prior hospitalization and not having been vaccinated against the influenza virus. There was no significant difference in the bivariate analysis regarding the use of 10-valent pneumococcal vaccine when comparing cases and controls.

**Table 2:** Distribution of the child-related factors and bivariate analysis for the risk of acquiring pneumonia

Variables		Cases (*n* = 407) N (%)	Controls (*n* = 407) N(%)	*p*-value	OR	95 % CI
Sex	Female	194 (47.7)	141 (34.6)		1	
	Male	213 (52.3)	266 (65.4)	<0.01	0.58	0.44–0.77
Age	≥1 year	241 (59.2)	324 (79.6)		1	
	<1 year	166 (40.8)	83 (20.4)	<0.01	2.68	1.97–3.67
Birth weight^a^	>2500 g	327 (87.9)	329 (85.5)		1	
	<2500 g	45 (12.1)	56 (14.5)	0.32	0.80	0. 53–1.23
Prematurity^b^	No	346 (91.0)	349 (89.3)		1	
	Yes	34 (9.0)	42 (10.7)	0.40	0.82	0.51–1.31
Respiratory disease /previous allergy	No	367 (90.2)	374 (91.9)		1	
	Yes	40 (9.8)	33 (8.1)	0.40	1.22	0.76–1.99
Previous hospitalization^c^	No	326 (81.7)	355 (87.2)		1	
	Yes	73 (18.3)	52 (12.8)	0.03	1.53	1.04–2.24
Exclusive Breastfeeding (4–6 months)^d^	Yes	203 (49.9)	212 (52.8)		1	
	No	204 (50.1)	192 (47.2)	0.53	1.09	0.83–1.44
Nutritional status (Weight-for-age Ratio) ^d^	(> -2 z score)	362 (89.2.)	383 (94.1)		1	
	(≤ -2 z score)	44 (10.8)	24 (5.9)	0.01	1.93	115–3.23
10-valent pneumococcal conjugated vaccine	Vaccinated	254 (62.4.)	258 (63.4)		1	
	unvaccinated	153 (37.6.)	149 (36.6)	0.77	1.04	0.78–1.38
Influenza virus vaccine	Vaccinated	172 (42.3)	289 (71.0)		1	
	unvaccinated	235 (57.7)	118 (29.0)	<0.01	3.34	2.50–4.48

Exclusive Breastfeeding when the child had been exclusively breastfed during 4 months

Nutritional status - according to a-2 z-score, the weight-for-age ratio was classified into two categories: very low or low weight for age and normal weight for age

10-valent pneumococcal conjugated vaccine children were considered to have been vaccinated if they had received at least two doses prior to their first birthday or one dose after their first birthday

Influenza virus vaccine children who were immunized according to the immunization card received: a) at least one dose if they received the prime vaccination previously and had been immunized with two doses Or b) two doses of the prime vaccination Children under 6 months of age and those over 6 months of age who had not been immunized were classified as unvaccinated

^a^Data missing in 35 cases (4.3 %);^b^Data missing 43cases (5.3 %);^c^Data missing in 8 cases (1.0 %); ^d^Data missing in 1 cases (0.1 %)

Table 3 shows the results of the bivariate analysis for the risk of CAP according to the socio demographic factors evaluated. All the factors evaluated were found to significantly increase the risk for CAP.

**Table 3:** Distribution of the sociodemographic factors and bivariate analysis for the risk of acquiring pneumonia

Variable		Cases (*n* = 407) N (%)	Controls (*n* = 407) N (%)	*p*-value	OR	95 % CI
Family income^*^	>1MS	156 (43.6)	201 (51.7)		1	
(minimum wage [MW])	≤1MS	202 (564)	188 (48.3)	0.03	1.38	1.04–1.84
Household crowding^†^	No	287 (721)	320 (86.5)		1	
	Yes	111 (27.9)	50 (13.5)	<0.01	2.47	1.70–3.58
Maternal age^‡^	≥19 years	369 (91.3)	389 (95.6)		1	
	<19 years	35 (8.7)	18 (4.4)	0.02	2.05	1.14–3.68
Maternal education^¶^(years of schooling)	≥11 years	151 (38.3)	196 (48.6)		1	
	<11 years	243 (61.7)	207 (51.4)	<0.01	1.52	1.15–2.02
Smoking in the home	No	285 (70.4)	321 (78.9)		1	
	Yes	120 (29.6)	86 (21.1)	<0.01	1.57	1.14–2.16
Maternal smoking	No	374 (91.9)	391 (96.1)		1	
	Yes	33 (8.1)	16 (3.9)	0.01	2.16	1.67–3.98

*Data missing in 67 cases (8.2 %); ^†^Data missing in 46 cases (5.6 %); ^‡^Data missing in 3cases (0.4 %); ^¶^Data missing in 19 cases (2.3 %)

Family income*(minimum wage MW]) - The minimum wage is the lowest remuneration that employers may legally pay to workers per month. During the period corresponding to the data collection the range was U$ 210.00 to 230.00

Household crowding† (≥2 individuals sleeping in the same room as the child

Table 4 shows all variables included in the first model of the multivariable analysis and the final mode. Only household crowding remained in the final model, doubling the likelihood of developing pneumonia (OR = 2.15). In contrast, being male was found to be a protective factor against pneumonia (OR = 0.53). In relation to vaccination compliance, not having been vaccinated against the influenza virus increased the likelihood of a child 1–59 months of age developing pneumonia by a factor of 3.59.

**Table 4:** Multivariate analysis of risk factors for community-acquired pneumonia in children aged 1–59 months

Variable	First Model			Final Model		
	Odds Ratio	*p*-value	95 % CI	Odds Ratio	*p*-value	95 % CI
Household crowding	1.79	0.01	1.14–2.80	2.15	<0.01	1.46–3.18
Male gender	0.52	<0.01	0.37–0.72	0.53	<0.01	0.39–0.72
Not immunized against the influenza virus	2.80	<0.01	1.82–4.30	3.59	<0.01	2.62–4.91
Age < 1 year	1.33	0.23	0.83–2.14	---	---	---
Exclusive breastfeeding	0.73	0.07	0.52–1.03	---	---	---
Undernutrition (weight-for-age ratio ≤ 2 z score)	1.50	0.21	0.79–2.85	---	---	---
Family income (≤1 minimum wage)	1.16	0.37	0.83–1.62	---	---	---
Maternal age	1.36	0.41	0.65–2.85	---	---	---
Maternal education	1.01	0.94	0.71–1.43	---	---	---
Smoking by household members	0.96	0.87	0.63–1.46	---	---	---
Maternal smoking	1.53	0.27	0.71–3.30	---	---	---
Constant	0.77	0.17	0.54–1.11	0.78	0.06	0.60–1.01

## Discussion

Brazil is among the 15 countries with the highest incidence of pneumonia, and this disease is the leading cause of death among children aged 1–4 years [2]. The introduction of the 10-valent pneumococcal conjugate vaccine in Brazil in 2010, the expanded use of influenza vaccine and the epidemiological transition resulting from improved socioeconomic conditions may have promoted changes in the risk factors for pneumonia in children between the ages of 1 month and 5 years. The implementation of the Unified Health System has improved primary access and allowed universal vaccine coverage [20].

No studies were previously conducted in Brazil to assess the risk factors for CAP in children under 5 years of age 2–3 years after the universal introduction of the 10-valent pneumococcal vaccine [10]. Additionally, during this time period, many social changes happened in the country [20]. The present study identified household crowding and not having been vaccinated against the influenza virus as risk factors for severe pneumonia, with male gender constituting a protective factor.

The definition of household crowding varies greatly [10]. In the present study, household crowding was defined as two or more individuals sleeping in the same room as the child, as this was the situation that most closely reflected the conditions reported by the parents or guardians of the children included in the study. Therefore, the permanence of this risk factor in the multivariate analysis enables us to conclude that, even following the improvement in economic conditions in the country, living conditions in homes with few rooms may facilitate the transmission of respiratory pathogens. These results are in agreement with a hospital-based case-control study [3] carried out in São Paulo; with a literature review [17] conducted in Brazil, including articles published between 1979 and 2004; and with a recent meta-analysis [10] published in 2013.

The role of sex as a risk factor for CAP remains unclear, and no consensus has been reached in the literature [28–30]. Males are more likely to develop lower respiratory tract infections. The greater resistance found in females can be explained by their enhanced Th1 immune response [29]. In the present study, male gender constituted a protective factor against pneumonia; however, regardless of the conflicting results in the literature, the present findings might be attributable to a selection bias, as there was a predominance of males in the control group resulting from the fact that elective surgeries in this age group are more common in boys.

As a means of preventing CAP, the WHO recommends that immunization programs include vaccines against measles, pertussis and influenza as well as *Haemophilus influenzae* type b and pneumococcal conjugate vaccines [31, 32]. The majority of case-control studies published in the literature were conducted prior to the introduction of the pneumococcal conjugate vaccine or were carried out in countries in which the vaccine was not routinely used [9, 10, 19, 21]. The measles vaccine was used as a reference for incomplete immunization in studies designed to evaluate risk factors for CAP [10].

In Brazil, the PCV10 was introduced in mid-2010. In 2013, an ecological study reported a 27.4 % reduction in the total number of cases of pneumonia in children under 2 years of age in Recife [14]. Therefore, the restricted vaccine coverage of only 60 % found in the present sample may constitute a concern. Nevertheless, data collection began in October 2010, and in addition to delayed vaccination, a fraction of the children in the age group evaluated were not included in the Ministry of Health’s immunization program, which was directed toward children 2–24 months of age. We did not observe the protective effect of the pneumococcal vaccine as would have been expected [25, 33–35]; however, the common characteristics between cases and controls should be taken into consideration. In both groups, the percentage of vaccinated children was similar, and the fact that no association was found between immunization and pneumonia suggests that the influence of other common factors in the two groups contributed to reducing the strength of the variables. The possibility of mixed etiology, especially co-infection with respiratory virus in cases of bacterial CAP has been well documented. Increasingly viral-viral interactions, bacterial and viral-bacterial-bacterial pathogenesis of respiratory infections are recognized [36–38]. Therefore, multiple etiology of pneumonia) - viruses, bacteria and even nonvaccine serotypes - can explain our findings.

In the multivariate model, the lack of vaccination against the influenza virus resulted in a significant 3.59-fold increase in the likelihood of developing pneumonia. In fact, up to 2013 (the data collection period), this vaccine was given to children 6 months to 2 years of age and only during the time of the year when the disease was prevalent, thus explaining the poor vaccine coverage. Studies reporting a protective effect of the influenza vaccine against pneumonia also referred to its protective role against co-infections by bacteria traditionally related to CAP, which may support the present findings [15, 36–38].

Age has been associated with a greater incidence of respiratory infections in the pediatric population, with children under 18 months of age being more vulnerable to CAP. Indeed, two-thirds of cases are concentrated in this age group [39]. In the present study, 41 % of the children in the case group were under 1 year of age, and in the control group, only 20.4 % were under 1 year of age. Although an age of ≤1 year was a significant risk factor in the bivariate analysis, which might be a selection bias of the control group, this factor did not remain in the final model.

A meta-analysis published in 2013 confirmed that undernutrition was a risk factor for pneumonia and showed a significant association between low weight for age and severe infection in developing countries. Nevertheless, no such association was found in the studies conducted in industrialized nations [10]. Considering that in the present sample low weight for age was found in only 10.8 % of the cases and 5.9 % of the controls, it may be assumed that Brazil, which is undergoing a demographic transition, has some of the characteristics of developed countries [20]. Another possible explanation for this finding is the homogeneity between the case and control groups insofar as socioeconomic conditions are concerned. Because body weight is strongly affected by socioeconomic level [17],when health-related factors were aggregated with factors associated with social conditions, the variable lost importance in the analysis [7].

Low birth weight, a factor that is preventable during the prenatal period, is associated with a greater risk of pneumonia and death [10, 40]. In the present study, the prevalence of low birth weight was similar to the reported national average and no statistically significant difference was found between the two groups.

Early weaning has been associated with a greater prevalence of CAP, with more severe cases and even death [19, 41, 42]. There was no difference between the two groups with respect to exclusive breastfeeding for 4–6-months, with approximately 50 % of children in both groups having been exclusively breastfed for that amount of time, a proportion similar to that estimated by the WHO for breastfeeding in Brazil [43].

Parents’ educational level, particularly maternal schooling, is inversely related to morbidity and mortality from pneumonia in childhood [4, 21, 44],as mothers with more years of schooling are presumably more capable of taking care of their children. The variable *whether the mother had completed high school* was used in the present evaluation in an attempt to make it as unlikely as possible that the positive variable would conceal cases of functional illiteracy, which, for healthcare-related purposes, is the same as complete illiteracy [45]. Similar to what was found in the present study, other investigators have also failed to find any association between these factors in multivariate analyses [10, 19, 42].

There is evidence of a causal relationship between poor socioeconomic conditions and pneumonia, with a greater frequency of CAP episodes in children from less privileged backgrounds [46]. In the present study, the likelihood of hospitalization because of pneumonia was greater when the family income was less than one minimum wage, a finding that has been reported by other investigators [6, 47]. Nevertheless, this association did not remain in the final model, reflecting the difficulty of assessing economic conditions based on salary alone, as the current social programs in the country may interfere with the real gain.

The observation that the percentage of adolescent mothers was higher in the case group compared to the control groups appears to corroborate the greater risk experienced by these children as a result of their mother’s immaturity and her unpreparedness to care for them [3]. Based on our understanding, the significance of this variable was weakened in the multivariate analysis because the intertwining relationships between socioeconomic factors and health are complex.

Exposure to cigarette smoking was an important preventable risk factor for admission to the hospital due to pneumonia in children under 5 years of age [48]. Other reports have emphasized the relevance of this association [49, 50]. In the present study, statistically significant differences were found between the groups in the bivariate analysis, both with respect to smoking in the home and maternal smoking; however, these differences were no longer present in the multivariate analysis. This finding is in agreement with the results of previous studies, which showed an inconsistent association [10]. Indoor air pollution was not assessed because in northeastern Brazil even low-income families do not use wood or coal for cooking, but butane GLP in their homes.

Of all the sociodemographic factors evaluated in the multivariate analysis, only household crowding was found to increase the likelihood of CAP. Therefore, this variable appears to summarize the combined effect of low income, poor maternal schooling and having been born to an adolescent mother, which may lead to different interpretations in epidemiological studies.

Brazil is a country of continental dimensions presenting socioeconomic and cultural differences between populations from different regions. We still have a significant economic inequality situation, so that in the same state, live children whose socioeconomic status is compatible with the family of highly developed regions and children in poverty. Ours results could be extrapolated to a group of children younger than five with the same socioeconomic status especially those in Northeast Brazil.

Case-control studies in general may be subject to recall, selection and registration biases, and to minimize these biases, we employed the incident cases strategy. To render the study feasible, the control group was selected from the community served by the hospital. The selection of controls focused on children who were “healthier”, i.e., patients without a previous history of pneumonia who had been admitted as outpatient basis for elective surgery. As in the selection of cases, the presence of any comorbidity that could contribute to CAP constituted an exclusion criterion, there by reducing the possibility of selection bias related to any primary disease.

## Conclusions

In conclusion, when the present results are compared with the findings of previous studies, it is clear that the risk factors for severe pneumonia have changed. The classical risk factors as undernutrition and mother’s education were not associated with CAP in our study population. Most likely, this fact is not only associated with the expansion of the vaccine program but also with the social improvements that have occurred in the country in recent years. However, these changes remain insufficient to overcome social inequalities, as shown by the findings related to household crowding. The possible protection offered by the influenza vaccine, as shown in this study, must be evaluated in etiological studies to assess the true role of this virus in the incidence of pneumonia in Brazil. Although the vaccine is offered only during the largest circulation period of the virus (autumn), the influenza virus circulates almost continuously throughout the year. Further studies on a wide range of possible risk factors that have not yet been evaluated in the community are required to clarify this question.
